# Supplementary material for: Prediction of spontaneous onset of labor at term (PREDICT study): Research protocol
Source: PLoS One. 2022 Jul 13;17(7):e0271065. doi: 10.1371/journal.pone.0271065 (PMC9278770; doi:10.1371/journal.pone.0271065)
Supplement: S1 Appendix — (DOCX) [file pone.0271065.s002.docx]

# Supporting Information

Appendix A

Specific Exclusion Criteria for Pregnolia® System according to the manufacturer’s recommendations (if any of these criteria is present, the measurement of cervical stiffness using Pregnolia® aspiration device will not be performed, but the patient will still be enrolled):

- Müllerian anomalies with two cervices
- Severe vaginal bleeding
- Cervical dilatation
- Known HIV infection
- Visible, symptomatic cervical or vaginal infections
- If one of the following conditions is present on the cervix at the 12 o’clock position:
  - Nabothian cyst
  - Cervical myomas
  - Cervical condylomas
  - Squamous intraepithelial lesion
  - Previous history of conization, LEEP2 or LLETZ3
  - Cervical endometriosis
  - Cervical tears
  - Cervical dysplasia
  - Large ectopy, for which it is not possible to find a suitable location near the ectopy where native tissue is present.
  - Large scar tissue, for which it is not possible to find a suitable location near the scar where native tissue is present.
