# Supplementary material for: Prediction of spontaneous onset of labor at term (PREDICT study): Research protocol
Source: PLoS One. 2022 Jul 13;17(7):e0271065. doi: 10.1371/journal.pone.0271065 (PMC9278770; doi:10.1371/journal.pone.0271065)
Supplement: S1 Protocol — (PDF) [file pone.0271065.s003.pdf]

Prediction of Spontaneous Onset of Labor at Term

Research legislation: Ordinance on human research with the exception of Clinical trials (HRO) [1].

Type of Research Project: Research project involving human subjects

Risk Categorization: A

Responsible:

Hôpitaux Universitaires de Genève
Catherine MCCAREY, MD (Principal investigator)
Cheffe de Clinique
Boulevard de la Cluse 30
1211 Genève, Suisse
+41 79 553 28 40
Catherine.Mccarey@hcuge.ch

Other collaborators:

Hôpitaux Universitaires de Genève
Begoña MARTÍNEZ DE TEJADA WEBER, MD, PhD
Cheffe de Service d'Obstétrique
Boulevard de la Cluse 30
1211 Genève, Suisse
+41 22 372 64 79
Begona.MartinezDeTejada@hcuge.ch

Mme. Véronique OTHENIN-GIRARD, Midwife
Hôpitaux Universitaires de Genève
Boulevard de la Cluse 30
1211 Genève, Suisse
+41 22 372 43 97
Veronique.Othenin-Girard@hcuge.ch

Mme. Antonina CHILIN, Midwife
Hôpitaux Universitaires de Genève
Boulevard de la Cluse 30
1211 Genève, Suisse
+41 79 553 23 02
Antonina.Chilin@hcuge.ch

Federico MIGLIORELLI, MD, PhD (Former Principal Investigator)
Study collaborator
Hôpitaux Universitaires de Genève
Boulevard de la Cluse 30
1211 Genève, Suisse
+34 607 31 32 57
femigliorelli@gmail.com

Collaborators from other centers:

Centre Hospitalier Universitaire Vaudois

David DESSEAUVE, MD, PhD
Principal investigator on site
Centre Hospitalier Universitaire Vaudois
Av Pierre-Decker 2
1011 Lausanne
+41 21 314 35 00
David.Desseauve@chuv.ch

David BAUD, MD, PhD
Centre Hospitalier Universitaire Vaudois
Av Pierre-Decker 2
1011 Lausanne
+41 21 314 35 00
David.Baud@chuv.ch

Yvan VIAL, MD, PhD
Centre Hospitalier Universitaire Vaudois
Av Pierre-Decker 2
1011 Lausanne
+41 21 314 35 00
Yvan.Vial@chuv.ch

Hélène LEGARDEUR, MD
Centre Hospitalier Universitaire Vaudois
Av Pierre-Decker 2
1011 Lausanne
+41 21 314 35 00
Helene.Legardeur@chuv.ch

Universitätsspital Basel

Franziska GEISSLER, MD, (Principal Investigator at site)
Spitalstrasse 21 /
4031 Basel, Suisse
+41 61 328 58 84
franziska.geissler@usb.ch

Anna CASPANI, MD (Local Investigator)
Spitalstrasse 21
4031 Basel, Suisse
+41 61 328 58 86
anna.caspani@usb.ch

Valeria FILIPPI, MD (Local Investigator)
Spitalstrasse 21
4031 Basel, Suisse

+41 61 328 58 90
valeria.filippi@usb.ch

Irene HÖSLI, MD,
Chief Physician Obstetrics and Antenatal Care
Spitalstrasse 21 /
4031 Basel, Suisse
+41 61 265 90 17
irene.hoesli@usb.ch

Cristina GRANADO (Study Coordinator at site, Midwife)
Spitalstrass 21
+41 61 328 66 90
studienhebamme@usb.ch

Doris MÜLLER BORER (Study Coordinator at site, Midwife)
Spitalstrass 21
+41 61 328 66 90
studienhebamme@usb.ch

PROTOCOL SIGNATURE FORM

Study Title ***Prediction of spontaneous onset of labor at term***

The project leader has approved the protocol version 5, dated 20.10.2021, and confirm hereby to conduct the project according to the protocol, the Swiss legal requirements [1,2], the current version of the World Medical Association Declaration of Helsinki [3] and the principles of Good Clinical Practice.

Project leader

Name: *Dr. Catherine Mc Carey*

Date: 20/10/2021 ________________ Signature: [redacted]

Sponsor:

Name: *Pre. Begoña Martínez de Tejada Weber*

Date: 20/10/2021 ________________ Signature: [redacted]

Project leader on site, Centre Hospitalier Universitaire Vaudois

Name: Dr.David Desseauve

Date: 20/10/2021____________________ Signature:

TABLE OF CONTENTS

TABLE OF CONTENTS 5
GLOSSARY OF ABBREVATIONS 7
1 BACKGROUND AND PROJECT RATIONALE 8
2 PROJECT OBJECTIVES AND DESIGN 11
2.1 Hypothesis, primary and secondary objectives 11
2.2 Primary and secondary endpoints 12
2.3 Project design 12
3 PROJECT POPULATION AND STUDY PROCEDURES 13
3.1 Project population, inclusion and exclusion criteria 13
3.2 Recruitment, screening and informed consent procedure (specific schedule of assessments for each patient is summarized in appendix 1) 14
3.3 Study procedures 14
3.4 Withdrawal and discontinuation 16
4 STATISTICS AND METHODOLOGY 17
4.1. Statistical analysis plan 17
4.2. Handling of missing data 17
5 REGULATORY ASPECTS AND SAFETY 18
5.1 Local regulations / Declaration of Helsinki 18
5.2 Notification of safety and protective measures (HRO Art. 20) 18
5.3 Serious events (HRO Art. 21) 18
5.4 Radiation 18
5.5 Amendments 18
5.6 End of project 18
5.7 Insurance 18
6 FURTHER ASPECTS 19
6.1 Overall ethical considerations 19
6.2 Risk-Benefit Assessment 19
6.3 Rationale for the inclusion of vulnerable participants 19
7 QUALITY CONTROL AND DATA PROTECTION 20
7.1 Quality measures 20
7.2 Data recording and source data 20
7.3 Confidentiality and coding 20
7.4 Retention and destruction of study data and biological material 20
8 FUNDING / PUBLICATION / DECLARATION OF INTEREST 20

9 REFERENCES 22
Appendix 1: Schedule of assessments 25

GLOSSARY OF ABBREVATIONS

BASEC	*Business Administration System for Ethical Committees*
CRF	*Case report form*
FOPH	*Federal Office of Public Health*
HRA	*Human Research Act*
HRO	*Ordinance on Human*

1 BACKGROUND AND PROJECT RATIONALE

Cesarean delivery rates are increasing worldwide, representing 18.6% of deliveries in 2016. However, this figure varies depending on the analyzed region, hence ranging from 6% to 27.2% between the least and most developed regions, respectively[5]. Despite being major surgery and associating far more maternal and neonatal risks[6] and lacking from a demonstrated benefit for both mother or the child[7], this trend has been continuously reproduced in different locations. The reasons for this increase are multifactorial and not yet well understood. They may include changes in maternal characteristics (as increasing maternal age for the first pregnancy, for example) and in professional practice styles, growing legal pressure, economic, organizational, social or cultural factors[8-10].

This situation has brought up concerns to the attention of many healthcare professionals and policy-makers, who started searching for strategies to stop (or even invert) the drift that these rates were taking. Thus, several strategies were adopted, including auditing or educational approaches, the design of multiple studies or the modification of the standard of care in many situations, such as the management of women with previous uterine scars or the mode of delivery in breech presentations[11,12].

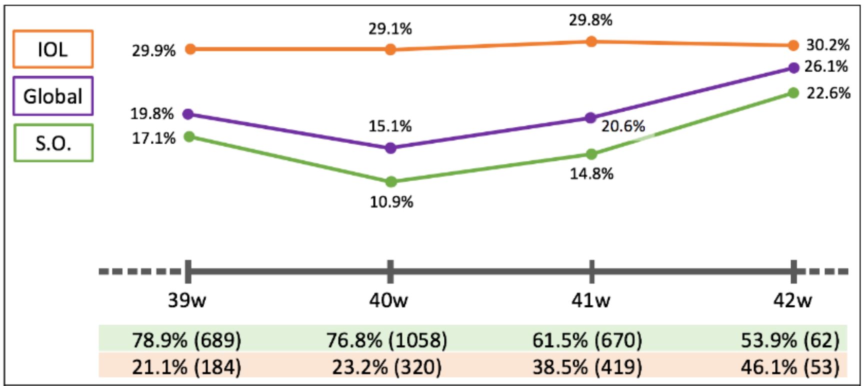

Cesarean delivery rates [induction of labor (IOL) vs. Spontaneous onset of labor (S.O.)]. Table below shows the number of spontaneously started labors (in green) and those who required induction (in orange) for different gestational ages. Data extracted from clinical records of deliveries at the maternity ward of the Hospital Clínic de Barcelona between June 2014 and December 2015. Unpublished.

Induction of labor (IOL) procedures (whose incidence also increased during the past years[13]) have been classically considered as responsible of a fraction of the augmented proportion of cesarean deliveries[14,15]. The example on the figure on the right seems to agree with this idea, as induced deliveries nearly duplicate the cesarean section rate of the spontaneously started labors. However, the evidence that supports the previous appreciation is based on retrospective observations which approached the issue as the contrast between the rate of cesarean deliveries in women who spontaneously started labor with those who required an IOL. Though, recent studies switched the perspective of the problem, prospectively comparing perinatal outcomes among women who were electively induced at certain gestational age to those who were expectantly managed (hence including women with spontaneous onset of labor, but also women induced for different causes lately). After assessing this strategy (i.e. elective IOL vs. expectant management) in several clinical scenarios, the obstetrical dogma claiming that "*IOL increased cesarean section rates*" was severely questioned.

Thus, the approach of electively inducing labor at certain gestational ages, in order to reduce the incidence of adverse obstetrical outcomes (where cesarean delivery is included), has been assessed in the settings of advanced maternal age[16], obesity[17] or, mostly, pregnancies over 41 weeks of gestation[18-20]. Most of these studies conclude that an active attitude towards IOL results, in these cases, in lower incidence of poorer results (including, in some of them, a diminution in intrauterine death rates), while the proportion of cesarean deliveries remains stable, or even decreases. The most recent example of this strategy has been the ARRIVE trial[21], where low-risk nulliparous women were randomly allocated to elective IOL at 39 weeks or to an expectant management group. The main outcome (a composite of neonatal results, including perinatal death) was similar for both groups, but a secondary analysis revealed that the proportion of cesarean deliveries was higher in those women assigned to the expectant management group than in the IOL cohort (22.2% vs. 18.6%, respectively, $p<0.001$). These results prompted some scientific societies, such as the Society of Maternal-Fetal Medicine (SMFM), to elaborate statements[22] where they claimed reasonable to offer elective IOL at 39 weeks of pregnancy to women who met ARRIVE eligibility criteria.

Although this recent evidence might support IOL as a method of improving perinatal outcomes, other issues must be addressed. For example, the $1.8^{0}/_{00}$ absolute reduction in perinatal mortality after elective IOL at 41 weeks (compared to the expectant attitude)[20] represents a requirement of more than 550

inductions in order to avoid one death. Consequently, this strategy would substantially increase the number of inductions in exchange of a discreet global benefit (perinatal mortality rate between 41 and 42 weeks is about 0.5$^{0}/_{00}$[23]). Following the results of the ARRIVE trial, 27 inductions would be needed in order to avoid one cesarean delivery. Evidence regarding cost-effectiveness and potential complications of this increase in IOL procedures is still lacking, hence the results of these studies have to been carefully interpreted before fitting them into current guidelines.

Furthermore, women's opinions in the matter of induction of labor must surely be considered. In fact, many women are uncomfortable with the idea of facing an IOL and more than half of them would rather not repeat the experience in subsequent pregnancies[24,25]. Therefore, any safety concern, especially when the benefit is limited, must be outweighed with maternal perceptions, choices and points of view.

An extremely polarized drift of all these circumstances might result in guidelines with universal recommendations of IOL at certain gestational ages, which will not be followed only by women who are against this policy and, due to their thoughts and beliefs, decide to *rebel* against the standard of care. So, to try to balance all these elements, it may be useful the development of a tool that aids in the decision-making process by differentiating:

(a) women who will spontaneously trigger their deliveries (thus theoretically profiting from an expectant management), hence followed-up according to current clinical guidelines
(b) women who will require IOL despite any period of expectancy, therefore with the need of a more individualized counseling and tailored prognosis concerning their options towards the end of their pregnancies.

The prediction of the spontaneous onset of labor becomes, then, the keystone of this approach. Many studies have tackled the idea of detecting features or biomarkers able to identify those women who will naturally start delivery and, mainly, three groups of variables have been analyzed:

- **Maternal characteristics** such as age[26,27], body mass index[26-28], parity[26,27,29], ethnicity[26] or Bishop score[30];
- **Ultrasonographic features**, including cervical length[26,29-34] or the presence of funneling[34];
- **Biochemical biomarkers** comprising the vaginal detection of fetal fibronectin[35,36].

Most of these studies have been performed to evaluate this issue in pregnancies arriving at 41 weeks[26,27,29,30], while a small sample of them were carried out at earlier gestational ages[31,32,34]. Among all the assessed variables, cervical length and body mass index were the most evaluated features and those that showed a higher association with spontaneous onset of labor. Regarding fetal fibronectin, it failed to discriminate women among the groups. However, it was scarcely analyzed in this field, as the efforts were mainly focused in preterm delivery. Concerning, Placental Alpha Microglobulin-1, it has not been yet evaluated for the prediction of term labor.

Although some variables, such as cervical length or body mass index, have shown certain association with spontaneous onset of labor, none of the authors have evaluated the association with a problem-solving approach, assessing the diagnostic accuracy of the found connections, thus without being able to succeed in transferring their research into clinical practice.

A biomarker that is gathering interest is the evaluation of cervical consistency. Traditionally included as one of the parameters evaluated in Bishop score, it has never achieved relevance as a predictor neither of preterm delivery nor of spontaneous onset of labor. One of its main flaws is its high subjectivity, which leads to a very low reproducibility. However, different authors have started to focus on cervical consistency as a marker of preterm delivery, assessed either by ultrasound (by means of the cervical consistency index[37]) or using aspiration devices, as the one developed Switzerland and commercialized by Pregnolia® AG (Information data sheet available as Appendix 2). Both of these techniques have shown that cervical consistency decreases with ongoing pregnancy[37,38], and the ultrasonographic evaluation has also been shown to be a potential predictor of preterm delivery[39] (but not of the result of induction of labor[40]), while Pregnolia® AG's device is currently being tested for these indications (SOFTCERVIX study - EUDAMED CIV-13-11-011694. Nevertheless, they have never been tested to evaluate the spontaneous

onset of labor. The Pregnolia System is used within the terms of its CE mark. It was certified by TUEV SUED 0123, CE CERTIFICATE NR. PROBE: G1S 106145 0004 Rev. 00, CE CERTIFICATE Nr, CONTROL UNIT: G2M 106145 0003 Rev .01

It is unlikely that a single variable will explain and perfectly classify women into the two groups of interest, as onset of labor is still a poorly known issue and probably related to many processes. Hence, the development of studies with the objective of prediction is mandatory, while the multifactorial characterization of the outcome needs to be addressed by the multiple and simultaneous evaluation of many of the features that may be related to the effect of interest, supported by an adequate statistical layout and design.

2 PROJECT OBJECTIVES AND DESIGN

2.1 Hypothesis, primary and secondary objectives

Hypothesis

The evaluation of different clinical features, together with the ultrasonographic assessment of local cervical conditions and the estimated fetal weight, combined with the biochemical determination of fetal Fibronectin and Placental Alpha Microglobulin-1 in vaginal secretions will provide the probability of spontaneous onset of labor during the remaining days of pregnancy, hence allowing the discrimination between those women who will spontaneously trigger their deliveries from those who will require an induction of labor.

Primary objectives

1. To evaluate the performance, at $38^{5/7}$-39 weeks of pregnancy, of different features in predictive models, designed to evaluate the probability of spontaneous onset of labor.

Secondary objectives

1. To analyze the individual accuracy of each of the evaluated characteristics at $38^{5/7}$-39 weeks of pregnancy to diagnose the onset of spontaneous labor.

2. The stablish the influence of each variable and the predictive models in the daily probability of spontaneous onset of labor after $38^{5/7}$-39 weeks of pregnancy.

3. To analyze the individual accuracy of each of the evaluated characteristics at 39 and 40 weeks of pregnancy to diagnose the onset of spontaneous labor within 1 or 2 weeks.

4. To evaluate the performance, at 39 and 40 weeks of pregnancy, of different features in predictive models, designed to evaluate the probability of spontaneous onset of labor within 1 or 2 weeks.

5. The stablish the influence of each variable and the predictive models in the daily probability of spontaneous onset of labor within 1 or 2 weeks after 39 and 40 weeks of pregnancy.

2.2 Primary and secondary endpoints

VARIABLE TYPE	VARIABLE AND SOURCE OF INFORMATION		
Main outcome (1) (logistic regression analysis)	Spontaneous onset of labor between 39 and $41^{3/7}$ weeks of pregnancy *vs.* Induction of labor between $41^{3/7}$ and 42 weeks of pregnancy due to gestational age		Review of clinical records
Main outcome (2) (survival analysis)	Gestational age at spontaneous onset of labor (days after last menstrual period)		Review of clinical records
Censorship	Induction of labor due to maternal or fetal indication, different from gestational age.<br>Prelabor rupture of membrane without spontaneous onset of labor after 12 hours of expectant management.		Review of clinical records
Predictors			
Clinical	Maternal age (years)<br>Maternal height (cm)<br>Closest to admission maternal weight (kg)<br>Previous vaginal deliveries<br>Previous cesarean deliveries<br>Expected delivery date		Review of clinical records
Clinical	Uterine cervical status (Bishop score)	Cervical digital exam	$38^{5/7}$-$39^{6/7}$ weeks
Ultrasonographic	Cervical length (mm)<br>Head to internal cervical os distance (mm)<br>Posterior cervical angle (degrees)<br>Cervical consistency index (%)	Transvaginal scan	$38^{5/7}$-$39^{6/7}$ weeks
Ultrasonographic	Estimated fetal weight (kg)	Transabdominal scan	$38^{5/7}$-$39^{6/7}$ weeks
Biochemical	Fetal Fibronectin (ng/mL) in vaginal secretion<br>Placental alpha macroglobulin 1 in vaginal secretion	Vaginal examination	$38^{5/7}$-$39^{6/7}$ weeks
Biophysical	Closing pressure (Cervical Stiffness Index, mbar) obtained by Pregnolia System as a marker of cervical stiffness	Cervical examination	$38^{5/7}$-$39^{6/7}$ weeks

2.3 Project design

Prospective international observational study with women enrolled at $38^{5/7}$-$39^{6/7}$ weeks of gestation, who will deliver in the Maternity Unit of the Hôpitaux Universitaires de Genève and in the Frauenklinik of Basel.

3 PROJECT POPULATION AND STUDY PROCEDURES

3.1 Project population, inclusion and exclusion criteria

ELIGIBILITY CRITERIA

Pregnant women at $38^{5/7}$-39 weeks of gestation who will deliver at the *Hôpitaux Universitaires de Genève*, Switzerland.

INCLUSION CRITERIA

- Alive fetus
- Singletons
- Cephalic presentation
- Intact membranes
- Gestational age ≥ $38^{5/7}$ weeks

EXCLUSION CRITERIA

- Fetal malformations
- Symptomatic uterine contractions
- Contraindication for vaginal delivery
- Medical indication of induction of labor or elective cesarean delivery
- Maternal desire to induce labor
- Maternal negative to enrollment
- Language barrier
- Inability to give consent

Specific Exclusion Criteria for Pregnolia System (if any of these criteria is present, the measurement of cervical stiffness using Pregnolia aspiration device will not be performed):

- Müllerian anomalies with two cervices
- Severe vaginal bleeding
- Cervical dilatation
- Known HIV
- Visible, symptomatic cervical or vaginal infections
- If one of the following conditions is present on the cervix at the 12 o'clock position:
  - Nabothian cyst
  - Cervical myomas
  - Cervical condylomas
  - Squamous intraepithelial lesion
  - Conization/LEEP2/LLETZ3
  - Cervical endometriosis
  - Cervical tears
  - Cervical dysplasia
  - Large ectopy, for which it is not possible to find a suitable location near the ectopy where native tissue is present
  - Large scar tissue, for which it is not possible to find a suitable location near the scar where native tissue is present.

<u>SAMPLE SIZE</u>

According to our data, approximately 78% of women will start their labor spontaneously before 41 weeks, meaning that 22% will have an induction of labor for medical reasons other than gestational age. Hence, considering 10 events (spontaneous onset of labor) for each of these 15 variables, and doubling this quantity for the elaboration of two different models, 13 participants will be required for each of these variables, hence 390 women are needed. Assuming a 10% rate of women with missing values in at least one predictor, 429 **patients** are needed.

3.2 Recruitment, screening and informed consent procedure (specific schedule of assessments for each patient is summarized in appendix 1)

Patient follow-up will be performed according to local guidelines, without introducing any modification due to the participation in the study. Besides current standard of care, clinical, ultrasonographic biochemical and biophysical parameters will be collected. Women will have the possibility to deny their participation or to withdraw from the study at any point. Likewise, their participation (or their denial) will not imply any change in the medical management of the pregnancy.

The recruitment, the standard of care and the data collection organization will be as follows:

(1) The pregnant woman will have her ordinary medical appointment at 34-38 weeks of gestation.
(2) Once finished the conventional medical consultation, eligibility criteria will be reviewed. If eligible, they will be offered to be enrolled in the study. After giving all the required information (which will include a specific information sheet), if the woman accedes to participate, <u>informed consent form</u> will be signed. Women will be encouraged to consider their decision and to ask all questions between this appointment and the following.

3.3 Study procedures

(1) If the woman consents her participation, she will be scheduled at $38^{5/7}$-$39^{6/7}$ weeks of pregnancy for assessment.
(2) During the visit, one of the researchers will perform:
    a. Review of clinical records and directed interview with the pregnant women in order to collect the required variables.
    b. Woman will be asked to empty her bladder
    c. Vaginal sample will be obtained using a sterile speculum and quantitative determination of fetal fibronectin and the qualitative determination of Placental Alpha Microglobulin-1 will be performed *in situ*, with the available commercialized kits for this purpose. The results of the tests will be registered, and the vaginal samples and the single-use medical devices will be discarded following hospital guidelines. We will also measure cervical stiffness using Pregnolia system device at this time. The Pregnolia System measurements are done according to the Instruction for use. The study team will be trained to ensure the right handling of the device. During the speculum application 3 consecutive cervical stiffness measurements at 12 o' clock position will be obtained (CSI 1, CSI 2, CSI 3) and reported in the CRF. Cervical stiffness measurement is only valid if the first measurement was successful.
    d. Transvaginal ultrasonographic scan will be performed and images will be recorded for off-line analysis.
    e. Transabdominal ultrasonographic scan will be performed to assess estimated fetal weight. The result will be blinded to the patient and it will only be registered in the case report form (to blind the result from the attending physicians).
(3) If any anomaly is detected during the ultrasound scan, the women will be referred and managed according to current guidelines.
(4) All results will be blinded to physicians in charge and to the participants (double blind). All extracted data will be recorded, and the researcher will ask the woman if she is willing to continue with her participation. If so, a further appointment will be scheduled at 40 weeks of pregnancy (minimum 1

week after the last visit), if labor does not start. Follow-up or the pregnancy will be performed in accordance to current standard of care, with her attending obstetrician or midwife.

a. From the 429 recruited participants, we expect that 60% will remain undelivered for the next consultation (257 women).
b. This means that, for all women, an initial set of data will be collected. However, more than half of them will have a second determination.
c. Therefore, for 429 women, 686 evaluations will be performed.

(5) Women initially approached at 40 weeks or pregnancy will be also eligible, as they will be included in the secondary stratified analysis.

(6) If labor does not spontaneously start during follow-up, in the absence of maternal or fetal conditions requiring early delivery, women will be scheduled for induction of labor between 41 and 42 weeks of pregnancy, following local guidelines.

(7) Clinical data, specific variables and perinatal outcomes will be recorded in an electronical case report form specifically designed for this purpose, adequately codified and anonymized, in order to avoid the possibility of identification and association of sensible data to concrete participants. All the information will be confidentially handled and will remain digitalized in an informatized database, password protected and only accessible by the main researcher. Data will be encoded for any kind of transport and all forms will be destroyed following current legislation.

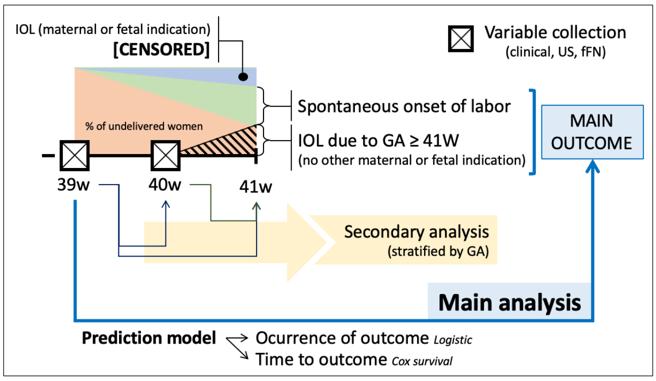

Study plan

	2019					2020											
	VIII	IX	X	XI	XII	I	II	III	IV	V	VI	VII	VIII	IX	X	XI	XII
Database creation																	
Elaboration of data collection form																	
Recruitment										Covid-19 Crisis							
Review of data, filtering and mistake correction																	
Multicentric meetings																	
Amendment submission																	

	2021												2022				
	I	II	III	IV	V	VI	VII	VIII	IX	X	XI	XII	I	II	III	IV	V
Recruitment					+												
Review of data, filtering and mistake correction																	
Kick-off meetings (online)																	
Monitoring visits																	
Wrap-up meetings (presential)																	
Image analysis																	
Statistical analysis																	
Conclusions																	
Communication of results																	

+ Start of multicenter collaboration

POSSIBLE LIMITATIONS

(1) *Women's refusal to participate*: all the suggested exams are commonly performed during pregnancy (transvaginal scan) or easily grabbed by routine vaginal exploration (fetal Fibronectin and Placental Alpha Microglobulin 1). Besides, as the participation does not imply any change in current standard of care, women are not exposed to subsequent risks due to their enrolment.
(2) *Bad quality images*: in order to avoid the interprofessional variability, the researcher team will be reduced to the minimum. Also, specific training will be offered in order to homogenize image acquisition criteria.
(3) *Women excluded due to findings in the ultrasound scans*: As ultrasonographic examination will be performed, there is a risk of finding anomalies in the scans which may prompt medical advice, leading to a possible exclusion of the participant from the study. If this happens, this situation will be recorded. However, to try to compensate this potential situation, sample size has been increased by 20%.

3.4 Withdrawal and discontinuation

Women may abandon the study at any time upon request, if they decide to withdraw their informed consent. In this case, they will be asked to decide about the destination of the information that has already been collected.

- If she decides not to consent any use of their data, the corresponding entrance will be removed from the database and all the paper documentation regarding their participation will be destroyed, complying with legal requirements.
- If she decides to consent the utilization of data, it will be kept as the rest of the data and the follow-up will be censored at the time of consent withdrawal.

It may also be possible that women discontinue their participation if a medical indication for elective cesarean delivery or induction of labor (different from gestational age) arises during the follow-up. In this case, censorship will be stablished at this point.

If perinatal outcomes are missing (due to delivery in another center, for example), follow-up will be considered up to the last medical registry and censored afterwards.

This potential loose of information has been considered in the design and the sample size has been adequately adjusted.

4 STATISTICS AND METHODOLOGY

4.1. Statistical analysis plan

MAIN ANALYSIS

The main outcome (spontaneous onset of labor vs. induction of labor between 41 and 42 weeks of pregnancy due to gestational age) will define two groups. The distribution of continuous or categorial variables among the units will be compared using Student's T test or Fischer's exact test, respectively. Two-sided significance level was set to 0.05.

A model of prediction of the binary outcome will be elaborated using stepwise backward and forward logistic regression analysis, including all analyzed features (gestational age at data collection will be included as an independent variable). To add or remove variables from the model, p-values of 0.05 or 0.10 will be used, respectively. Goodness-of-fit of the model will be evaluated using Hosmer and Lemeshow test and, if multiple models are achieved, the best one will be selected searching parsimony and following Akaike's Information Criterion. Area under the Receiver-Operating Characteristics curve will be used to assess predictive ability of the model. This value will be also calculated using bootstrapping resampling methods (1000 repetitions) to internally validate the results. As only a subset of the sample will have the measurement of cervical stiffness using Pregnolia device (as this assessment was lately introduced), a secondary analysis (following the same principles of the main analysis) will be performed. A correlation analysis will be carried out comparing cervical consistency index evaluated by ultrasound with cervical stiffness assessed using the aspiration device, as both measurement evaluate the same biophysical characteristic of the cervix.

The probability of spontaneous onset of labor for each day after the analysis will be evaluated using survival regression models. The data will be described using Kaplan-Meier's non-parametrical survival function and the predictive model will be estimated using stepwise backward and forward Cox's regression (proportional hazards model). As mentioned before, data regarding women whose labors have been induced due to maternal or fetal indication (different from gestational age) or with prelabor rupture of membrane without spontaneous onset of labor after 12 hours of expectant management will be censored at the time of admission for these causes.

SECONDARY ANALYSIS (STRATIFICATION BY GESTATIONAL AGE)

Besides the analysis that guides the design of the study, both logistic regression and survival analysis will be performed as follows:

(1) For data collected at $38^{5/7}$-$39^{6/7}$ weeks of gestation: Probability of outcome in 7 and 14 days after data collection.
(2) For data collected at 40 weeks ($40^{0/7}$-$40^{6/7}$) of gestation: Probability of outcome in 7 and 10 days after data collection.

The same censorship criteria will be applied.

All statistical analysis will be performed using Stata 16.1 software (StataCorp, Texas, USA).

4.2. Handling of missing data

As it has been mentioned in the sample size estimation, the required number of participants has been increased in order to compensate potential loss, mainly due to the arising of medical complications that may lead to elective cesarean deliveries or to an induction of labor due to causes different from gestational age. We consider that delivering outside the Hospital is an unlikely event, as the eligibility criteria specify that women plan to deliver within the center.

Missing data in the predictive variables will lead to the exclusion of the participant from the regression models. Hence, a 10% increase in sample size has been estimated to compensate this issue.

5 REGULATORY ASPECTS AND SAFETY

5.1 Local regulations / Declaration of Helsinki

This research project will be conducted in accordance with the protocol, the Declaration of Helsinki [3], the principles of Good Clinical Practice, the Human Research Act (HRA) and the Human Research Ordinance (HRO) [1] as well as other locally relevant regulations. The Project Leader acknowledges his responsibilities as both the Project Leader and the Sponsor.

5.2 Notification of safety and protective measures (HRO Art. 20)

The project leader is promptly notified (within 24 hours) if immediate safety and protective measures have to be taken during the conduct of the research project. The Ethics Committee will be notified via BASEC of these measures and of the circumstances necessitating them within 7 days.

5.3 Serious events (HRO Art. 21)

If a serious event occurs, the research project will be interrupted, and the Ethics Committee notified on the circumstances via BASEC within 7 days according to HRO Art. 21[1]. The manufacturer of the Pregnolia Device will be informed about Serious Adverse Events or any related adverse events (Adverse Device Effect).

5.4 Radiation

No radioactive sources will be used during the development of the research project. Ultrasound examination will be performed following ALARA (As Low As Reasonably Acceptable) criteria.

5.5 Amendments

Substantial changes to the project set-up, the protocol and relevant project documents will be submitted to the Ethics Committee for approval according to HRO Art. 18 before implementation. Exceptions are measures that have to be taken immediately in order to protect the participants.

5.6 End of project

Upon project termination, the Ethics Committee is notified within 90 days. Data will remain encoded and anonymized and all forms will be destroyed following current legislation. Ultrasonographic images will be deleted from hard drive devices. Health-related data will be anonymized upon termination of data analysis and safely stored.

5.7 Insurance

As it is a study category A, no insurance is needed.

---

[1] A serious event is defined as any adverse event where it cannot be excluded, that the event is attributable to the sampling of biological material or the collection of health-related personal data, and which:
a. requires inpatient treatment not envisaged in the protocol or extends a current hospital stay;
b. results in permanent or significant incapacity or disability; or
c. is life-threatening or results in death.

6 FURTHER ASPECTS

6.1 Overall ethical considerations

Women's opinions in the matter of induction of labor, in the current clinical setting, must be considered. In fact, many women are uncomfortable with the idea of facing an IOL and more than half of them would rather not repeat the experience in subsequent pregnancies. Therefore, any safety concern, especially when the benefit is limited, must be outweighed with maternal perceptions, choices and points of view. Hence, guidelines with universal recommendations of IOL at certain gestational ages might not be followed by women who are against this policy. So, the development of a predictive tool might offer data that will aid in the decision-making process.

The participation in the study will represent for women time effort and the need to subject to extra ultrasonographic explorations. However, these have already demonstrated their lack of deleterious effect on the pregnancy, therefore no harm is expected over maternal or fetal health.

Data will be collected in a form specifically designed for this purpose, adequately codified and anonymized, in order to avoid the possibility of identification and association of sensible data to concrete participants. All the information will be confidentially handled and will remain digitalized in an informatized database, password protected and only accessible by the main researcher. Data will be encoded for any kind of transport and all forms will be destroyed following current legislation. Any incidental finding during analysis will be carefully evaluated and only pondered in the conclusions as *hypothesis-generator*, considering the multiple biases introduced by the assumption of true findings after studies which were not designed for these purposes.

The study will be carried out following the *Good Clinical Practice*, the principles of the *Declaration of Helsinki* and the *Medical Research Involving Human Subjects Act (WHO).*

Given that the nature of the study is non-experimental (observational) and that the security of the interventions has been widely evaluated during pregnancy, women are not exposed to any risk due to their participation in this research. Likewise, different ethical aspects have been assessed, without finding any conflict open to modifications. All participants will be adequately informed, both orally and in writing, and the main research will remain at their disposal to answer any question that they may find necessary. They will also sign the informed consent form before performing any intervention and they will have the possibility to withdraw from the study at any point, without changes in medical standard of care. They will also have access to the verification of the personal information kept by the researchers and will be notified of the results of the study.

6.2 Risk-Benefit Assessment

Apart from the benefit for current scientific knowledge and the potential impact in future management of pregnancies, we do not expect immediate benefits for the participants. However, the study does not represent any risk to women, as the explorations that will be carried out are usually performed in current standard of care. Also, as all data will remain anonymized, there is no risk of personal data diffusion and participant identification if unauthorized data access occurs. Therefore, the risk-benefit balance is clearly leaning to the second.

6.3 Rationale for the inclusion of vulnerable participants

The inclusion of pregnant women is mandatory for studying the situations involving the delivery. The risk for the participants is minimal, as the interventions are currently performed during pregnancy and have already demonstrated its safety both for the mother and the fetus. The results of the research will have an impact over the management of pregnant women.

7 QUALITY CONTROL AND DATA PROTECTION

7.1 Quality measures

Data will be collected by the researchers in specifically designed electronic Case Report Forms, assuring the maximum number of closed questions. All data will be double entered to the database only by the researchers who were trained to handle it, with adequate knowledge in data management and confidentiality. One of the researchers -who was not in charge of data recording- will evaluate data entry by aleatorily selecting a 10% of the forms and verify their correct entrance into the database. Also, inadequate or missing values will be periodically assessed, in order to evaluate data quality. This will be periodically performed at all recruiting centers.

For quality assurance the Ethics Committee may visit the research sites. Direct access to the source data and all project related files and documents must be granted on such occasions.

7.2 Data recording and source data

Data will be recorded using electronic Case Repot Forms using Redcap® software. Team researchers will be specifically trained to adequately manage this information using this platform and each center will be responsible of entering the data they produce.

Data will be retrieved from three different sources:

- Biochemical results will be immediately obtained after processing the samples and they will be recorded by the researchers, completely blinding clinicians and participants from results.
- Ultrasonographic images will be recorded in Digital Imaging and Communications in Medicine (DICOM) format and analyzed off-line using specific software.
- Maternal characteristics and perinatal results will be collected from clinical records.

7.3 Confidentiality and coding

Project data will be handled with uttermost discretion and is only accessible to authorized personnel who require the data to fulfil their duties within the scope of the research project. On the CRFs and other project specific documents, participants are only identified by a unique participant number.

Participant identification lists, and data will be stored in Redcap® databases, which will be password protected and only accessible by authorized researchers. Each center will have its own access to the software, with access only to the information that they have introduced. Only the main researchers will have access to the full set of data.

7.4 Retention and destruction of study data and biological material

Biological samples (vaginal swabs) will be destroyed after obtaining the result of the analysis, which is performed immediately after its collection. Data will be encoded for any kind of transport and all forms will be destroyed following current legislation, 10 years after finishing recruitment.

8 FUNDING / PUBLICATION / DECLARATION OF INTEREST

The developers of fetal Fibronectin determination kits (Hologic®) will provide them, along with the analyzers at no cost for the researchers. Funding and supplies support will be asked to the developers of Placental alpha macroglobulin 1 determination kits (Qiaogen®).

Regarding the collaboration with the enterprise “Pregnolia AG®”, which will be providing the device for the measurement of cervical stiffness, a collaboration contract has been established. One Pregnolia Control Unit , as well as 240 disposable Pregnolia Probes, will be provided free of charge for the conduct of the research.

Both enterprises have been informed that the study protocol will be published, as well as the conclusions of the research, whatever are the results. The design of the study, the data collection, analysis and publication are an exclusive responsibility of the researchers and no interference from the enterprises is expected. Data will not be shared, and the companies will only know the results once the analysis has finished.

The researchers are workers payed by the institutions involved in the study and will not receive any compensation for participating in the study. Once collaboration contracts are stablished with any of these two companies, SwissEthics will be immediately informed.

The contract regarding multicentric collaboration is attached to this protocol.

9 REFERENCES

1. Ordinance on Human Research with the Exception of Clinical trials (HRO) http://www.admin.ch/opc/en/classified-compilation/20121177/201401010000/810.301.pdf
2. Human Research Act (HRA) http://www.admin.ch/opc/en/classified-compilation/20121176/201401010000/810.305.pdf
3. Declaration of Helsinki (http://www.wma.net/en/30publications/10policies/b3/index.html)

STROBE statement (http://www.jclinepi.com/article/S0895-4356(07)00436-2/pdf)1. Perissinotti A, Paredes P, Vidal-Sicart S, Torné A, Albela S, Navales I, Martínez-Román S, Pahisa J, Pons F. Use of SPECT/CT for improved sentinel lymph node localization in endometrial cancer. *Gynecol Oncol.* 2013;129(1):42-48. doi:10.1016/j.ygyno.2013.01.022.

2. Barlin JN, Zhou Q, St. Clair CM, Iasonos A, Soslow RA, Alektiar KM, Hensley ML, Leitao MM, Barakat RR, Abu-Rustum NR. Classification and regression tree (CART) analysis of endometrial carcinoma: Seeing the forest for the trees. *Gynecol Oncol.* 2013;130(3):452-456. doi:10.1016/j.ygyno.2013.06.009.
3. Savas N. Gastrointestinal endoscopy in pregnancy. *World J Gastroenterol.* 2014;20(41):15241-15252. doi:10.3748/wjg.v20.i41.15241.
4. Prachayakul V, Aswakul P. Endoscopic ultrasound-guided interventions in special situations. *World J Gastrointest Endosc.* 2016;8(2):104. doi:10.4253/wjge.v8.i2.104.
5. Betrán AP, Ye J, Moller A-B, Zhang J, Gülmezoglu AM, Torloni MR. The Increasing Trend in Caesarean Section Rates: Global, Regional and National Estimates: 1990-2014. *PLoS One.* 2016;11(2):e0148343. doi:10.1371/journal.pone.0148343.
6. Gregory KD, Jackson S, Korst L, Fridman M. Cesarean versus vaginal delivery: Whose risks? whose benefits? *Am J Perinatol.* 2012;29(1):7-18. doi:10.1055/s-0031-1285829.
7. Lumbiganon P, Laopaiboon M, Gülmezoglu AM, Souza JP, Taneepanichskul S, Ruyan P, Attygalle DE, Shrestha N, Mori R, Hinh ND, Bang HT, Rathavy T, Chuyun K, Cheang K, Festin M, Udomprasertgul V, Germar MJ V., Yanqiu G, Roy M, Carroli G, Ba-Thike K, Filatova E, Villar J. Method of delivery and pregnancy outcomes in Asia: the WHO global survey on maternal and perinatal health 2007-08. *Lancet.* 2010;375(9713):490-499. doi:10.1016/S0140-6736(09)61870-5.
8. Zwecker P, Azoulay L, Abenhaim HA. Effect of fear of litigation on obstetric care: A nationwide analysis on obstetric practice. *Am J Perinatol.* 2011;28(4):277-283. doi:10.1055/s-0030-1271213.
9. Linton A, Peterson MR, Williams T V. Effects of maternal characteristics on cesarean delivery rates among U.S. Department of Defense Healthcare Beneficiaries, 1996-2002. *Birth.* 2004;31(1):3-11. doi:10.1111/j.0730-7659.2004.0268.x.
10. Lin H-C, Xirasagar S. Institutional Factors in Cesarean Delivery Rates: Policy and Research Implications. *Obstet Gynecol.* 2004;103(1):128-136. doi:10.1097/01.AOG.0000102935.91389.53.
11. Chaillet N, Dumont A. Evidence-based strategies for reducing cesarean section rates: A meta-analysis. *Birth.* 2007;34(1):53-64. doi:10.1111/j.1523-536X.2006.00146.x.
12. Agosta LJ, Johnson C. Implementing Interventions Aimed at Reducing Rates of Cesarean Birth. *Nurs Womens Health.* 2017;21(4):260-273. doi:10.1016/j.nwh.2017.06.006.
13. Osterman MJK, Martin JA. Recent declines in induction of labor by gestational age. *NCHS Data Brief.* 2014;(155):1-8. doi:10.1017/CBO9781107415324.004.
14. Kiesewetter B, Lehner R. Maternal outcome monitoring: Induction of labor versus spontaneous onset of labor-a retrospective data analysis. *Arch Gynecol Obstet.* 2012;286(1):37-41. doi:10.1007/s00404-012-2239-0.
15. Hoffman MK, Vahratian A, Sciscione AC, Troendle JF, Zhang J. Comparison of labor progression between induced and noninduced multiparous women. *Obstet Gynecol.* 2006;107(5):1029-1034. doi:10.1097/01.AOG.0000210528.32940.c6.
16. Knight HE, Cromwell DA, Gurol-Urganci I, Harron K, van der Meulen JH, Smith GCS. Perinatal mortality associated with induction of labour versus expectant management in nulliparous women aged 35 years or over: An English national cohort study. *PLoS Med.* 2017;14(11):e1002425. doi:10.1371/journal.pmed.1002425.
17. Gibbs Pickens CM, Kramer MR, Howards PP, Badell ML, Caughey AB, Hogue CJ. Term Elective Induction of Labor and Pregnancy Outcomes Among Obese Women and Their Offspring. *Obstet Gynecol.* 2018;131(1):12-22. doi:10.1097/AOG.0000000000002408.

18. Caughey AB, Stotland NE, Washington AE, Escobar GJ. Who is at risk for prolonged and postterm pregnancy? *Am J Obstet Gynecol*. 2009;200(6):683.e1-683.e5. doi:10.1016/j.ajog.2009.02.034.

19. Caughey AB, Sundaram V, Kaimal AJ, Gienger A, Cheng YW, McDonald KM, Shaffer BL, Owens DK, Bravata DM. Systematic Review: Elective Induction of Labor Versus Expectant Management of Pregnancy. *Ann Intern Med*. 2009;151(4):252-263. doi:10.7326/0003-4819-151-4-200908180-00007.

20. Middleton P, Shepherd E, Crowther C. Induction of labour for improving birth outcomes for women at or beyond term (Review). *Cochrane Database Syst Rev*. 2018;(5):Art. No.: CD004945. doi:10.1002/14651858.CD004945.pub4.

21. Grobman WA, Rice MM, Reddy UM, Tita ATN, Silver RM, Mallett G, Hill K, Thom EA, El-Sayed YY, Perez-Delboy A, Rouse DJ, Saade GR, Boggess KA, Chauhan SP, Iams JD, Chien EK, Casey BM, Gibbs RS, Srinivas SK, Swamy GK, Simhan HN, Macones GA. Labor Induction versus Expectant Management in Low-Risk Nulliparous Women. *N Engl J Med*. 2018;379(6):513-523. doi:10.1056/NEJMoa1800566.

22. Society of Maternal-Fetal (SMFM) Publications Committee. SMFM Statement on Elective Induction of Labor in Low-Risk Nulliparous Women at Term: The ARRIVE Trial. *Am J Obstet Gynecol*. 2018:3-6. doi:10.1016/j.ajog.2018.08.009.

23. Zizzo AR, Kirkegaard I, Pinborg A, Ulbjerg N. Decline in stillbirths and perinatal mortality after implementation of a more aggressive induction policy in post-date pregnancies: a nationwide register study. *Acta Obstet Gynecol Scand*. 2017;96(7):862-867. doi:10.1111/aogs.13113.

24. Schwarz C, Gross MM, Heusser P, Berger B. Women's perceptions of induction of labour outcomes: Results of an online-survey in Germany. *Midwifery*. 2016;35:3-10. doi:10.1016/j.midw.2016.02.002.

25. Heimstad R, Romundstad PR, Hyett J, Mattsson L-Å, Salvesen KÅ. Women's experiences and attitudes towards expectant management and induction of labor for post-term pregnancy. *Acta Obstet Gynecol Scand*. 2007;86(8):950-956. doi:10.1080/00016340701416929.

26. Rao A, Celik E, Poggi S, Poon L, Nicolaides KH. Cervical length and maternal factors in expectantly managed prolonged pregnancy: Prediction of onset of labor and mode of delivery. *Ultrasound Obstet Gynecol*. 2008;32(5):646-651. doi:10.1002/uog.6211.

27. Marquette GP, Hutcheon JA, Lee L. Predicting the Spontaneous Onset of Labour in Post-Date Pregnancies: A Population-Based Retrospective Cohort Study. *J Obstet Gynaecol Canada*. 2014;36(5):391-399. doi:10.1016/S1701-2163(15)30584-3.

28. Hermesch AC, Allshouse AA, Heyborne KD. Body Mass Index and the Spontaneous Onset of Parturition. *Obstet Gynecol*. 2016;128(5):1033-1038. doi:10.1097/AOG.0000000000001678.

29. Vankayalapati P, Sethna F, Roberts N, Ngeh N, Thilaganathan B, Bhide A. Ultrasound assessment of cervical length in prolonged pregnancy: Prediction of spontaneous onset of labor and successful vaginal delivery. *Ultrasound Obstet Gynecol*. 2008;31(3):328-331. doi:10.1002/uog.5254.

30. Strobel E, Sladkevicius P, Rovas L, De Smet F, Dejin Karlsson E, Valentin L. Bishop score and ultrasound assessment of the cervix for prediction of time to on set of labor and time to delivery in prolonged pregnancy. *Ultrasound Obstet Gynecol*. 2006;28(3):298-305. doi:10.1002/uog.2805.

31. Ramanathan G, Yu C, Osei E, Nicolaides KH. Ultrasound examination at 37 weeks' gestation in the prediction of pregnancy outcome: The value of cervical assessment. *Ultrasound Obstet Gynecol*. 2003;22(6):598-603. doi:10.1002/uog.913.

32. Rizzo G, Aiello E, Pietrolucci ME, Arduini D. Ultrasonographic assessment of cervical length in pregnancies scheduled for a cesarean delivery: Prediction of early spontaneous onset of labor. *J Perinat Med*. 2015;2015. doi:10.1515/jpm-2015-0238.

33. Meijer-Hoogeveen M, Van Holsbeke C, Van Der Tweel I, Stoutenbeek P, Visser GHA. Sonographic longitudinal cervical length measurements in nulliparous women at term: Prediction of spontaneous onset of labor. *Ultrasound Obstet Gynecol*. 2008;32(5):652-656. doi:10.1002/uog.5291.

34. Bayramoglu Ö, Arslan M, Yazici FG, Erdem A, Erdem M, Bayramoglu K, Camdeviren H. Prediction of spontaneous onset of labor at term: The role of cervical length measurement and funneling of internal cervical os detected by transvaginal ultrasonography. *Am J Perinatol*. 2005;22(1):35-39. doi:10.1055/s-2004-837268.

35. Kiss H, Ahner R, Hohlagschwandtner M, Leitich H, Husslein P. Fetal fibronectin as a predictor of term labor: A literature review. *Acta Obstet Gynecol Scand*. 2000;79(1):3-7. doi:10.1034/j.1600-0412.2000.079001003.x.

36. Rozenberg P, Goffinet F, Hessabi M. Comparaison du score de Bishop, de la mesure échographique de la longueur du col, et du dosage de la fibronectine dans la prédiction du délai et de la voi d'accouchement à

terme. *Bull Acad Natle Méd*. 1999;183(3):589-600.

37. Parra-Saavedra M, Gómez L, Barrero A, Parra G, Vergara F, Navarro E. Prediction of preterm birth using the cervical consistency index. *Ultrasound Obstet Gynecol*. 2011;38(1):44-51. doi:10.1002/uog.9010.

38. Badir S, Mazza E, Zimmermann R, Bajka M. Cervical softening occurs early in pregnancy: Characterization of cervical stiffness in 100 healthy women using the aspiration technique. *Prenat Diagn*. 2013;33(8):737-741. doi:10.1002/pd.4116.

39. Migliorelli F, Baños N, Angeles MA, Rueda C, Salazar L, Gratacós E, Palacio M. Clinical and sonographic model to predict cesarean delivery after induction of labor at term. *Fetal Diagn Ther*. 2019;46(2). doi:10.1159/000493343.

40. Migliorelli F, Rueda C, Angeles MA, Baños N, Posadas DE, Gratacós E, Palacio M. Cervical consistency index and risk of Cesarean delivery after induction of labor at term. *Ultrasound Obstet Gynecol*. 2019;53(6):798-803. doi:10.1002/uog.20152.

Appendix 1: Schedule of assessments

Time (gestational age)	*34-38 weeks*	*$38^{5/7}$-$39^{6/7}$ weeks*	*Delivery*	*1 week after delivery*
Visit	Information	Visit		
Check eligibility criteria	+			
Oral and written Information	+			
Written consent		+		
Check inclusion/ exclusion criteria		+		
Medical history		+		
Participant Characteristics		+		
Ultrasonographic examination		+		
Vaginal sampling and testing for fetal Fibronectin and Placental Alpha Microglobulin 1		+		
Collection of perinatal outcomes				+
